# Acupuncture regulates mitochondrial homeostasis in traumatic brain injury: current evidence, mechanistic hypotheses, and translational challenges

**DOI:** 10.1186/s13020-026-01481-2

**Published:** 2026-08-14

**Authors:** Guanglei Li, Luxi Cao, Sisi Zhao, Yingyu Fang, Zhentao Peng, Feidan Deng, Peng Qing, Mingmin Zhu, Yimin Zhang, Shujun Lin

**Affiliations:** 1 College of Traditional Chinese Medicine, Jinan University, Guangzhou, 510632 China; 2 South China Research Center for Acupuncture and Moxibustion, Guangzhou University of Chinese Medicine, Guangzhou, 510006 China; 3 Clinical Medical College of Acupuncture Moxibustion and Rehabilitation, Guangzhou University of Chinese Medicine, Guangzhou, 510006 China; 4 The First Affiliated Hospital of Jinan University, Guangzhou, 510630 China

**Keywords:** Acupuncture, Electroacupuncture, Traumatic brain injury, Mitochondrial homeostasis, Mitophagy, Neuroprotection

## Abstract

**Background:**

Traumatic brain injury (TBI) is associated with high morbidity, disability, and long-term neurological sequelae. Mitochondrial dysfunction is a central component of secondary injury after TBI, contributing to impaired energy metabolism, oxidative stress, calcium dysregulation, neuroinflammation, and neuronal apoptosis.

**Main body:**

This review summarizes current evidence regarding acupuncture-mediated regulation of mitochondrial homeostasis after TBI, focusing on structural homeostasis, quantitative homeostasis, and functional/metabolic homeostasis. We distinguish direct evidence from TBI models, indirect evidence from related brain injury models, and hypothesis-generating mechanisms. Particular attention is given to mitochondrial dynamics, mitochondrial biogenesis, mitophagy, energy metabolism, oxidative stress, calcium signaling, mitochondrial membrane potential, apoptosis, intercellular mitochondrial transfer, and putative upstream neural and humoral pathways.

**Conclusion:**

Current preclinical evidence suggests that acupuncture, particularly electroacupuncture, may influence mitochondrial homeostasis after TBI through multidimensional and context-dependent mechanisms. However, direct TBI-specific evidence remains limited, and several proposed mechanisms require further validation before acupuncture-mediated mitochondrial regulation can be considered a clinically established therapeutic strategy.

## Introduction

Traumatic brain injury (TBI), defined as structural or functional brain damage caused by external mechanical force, remains a leading global cause of neurological morbidity and disability. An estimated 69 million individuals sustain TBI worldwide each year, with an all-severity incidence of approximately 939 cases per 100,000 population [1]. In the Global Burden of Disease 2021 analysis, TBI accounted for 20.84 million incident cases, 37.93 million prevalent cases, and 5.48 million years lived with disability globally [2]. These findings highlight the persistent burden of TBI and the need to identify modifiable mechanisms that contribute to long-term neurological sequelae. The pathological process of TBI is typically divided into primary and secondary injury. Primary injury consists of immediate mechanical events such as impact loading, shear stress, membrane disruption, vascular damage, and diffuse axonal injury, whereas secondary injury involves delayed cascades including oxidative stress, excitotoxicity, mitochondrial dysfunction, inflammatory responses, blood–brain barrier disruption, and cell death [1–3]. Because currently available clinical interventions cannot fully prevent long-term neurological sequelae, identifying modifiable molecular targets within the secondary injury cascade remains an important research priority.

Mitochondria, known as the "powerhouses" for neuronal survival and functional maintenance, are responsible not only for the synthesis of adenosine triphosphate (ATP) but also for the regulation of critical physiological processes including oxidative stress, calcium homeostasis, and apoptosis [4]. The homeostasis of these double-membrane organelles is essential for neuronal function, whereas TBI induces profound spatiotemporal disruption of mitochondrial homeostasis [5, 6]. Specifically, mechanical damage, ischemia-hypoxia, and inflammatory responses triggered by external impact directly impair mitochondrial structure, leading to dynamic imbalance, autophagic dysfunction, inhibited biosynthesis, and excessive activation of oxidative stress. These mitochondrial dysfunctions further exacerbate neuronal injury and neurological deficits. Mitochondrial homeostasis imbalance is thus recognized as a key event in TBI-induced secondary injury, which can subsequently cause reactive oxygen species (ROS) accumulation, ATP depletion, and neuronal apoptosis [4, 7].

Acupuncture is a non-pharmacological intervention widely used in neurological rehabilitation. In preclinical TBI models, acupuncture or electroacupuncture has been reported to improve neurological function scores and reduce histopathological injury [8–10], while limited clinical evidence suggests potential benefit for cognitive recovery in mild TBI [11]. Nevertheless, the current evidence base is dominated by animal studies, and robust clinical trials with standardized stimulation protocols and mechanistic biomarkers remain scarce. This review therefore does not aim to claim that acupuncture has a validated mitochondrial mechanism in clinical TBI. Instead, it critically evaluates direct TBI evidence and indirect evidence from related brain injury models, with clear separation between established observations, supportive findings, and hypothesis-generating mechanisms. Since several mechanistic studies are derived from ischemic stroke or intracerebral hemorrhage models, these findings are used only when they illuminate shared secondary injury processes such as oxidative stress, mitochondrial dysfunction, and neuroinflammation. They should not be interpreted as definitive evidence for TBI-specific mechanisms.

### Literature search and evidence grading

We performed a targeted narrative literature search, rather than a systematic review or meta-analysis, in PubMed, Web of Science Core Collection, CNKI, and Wanfang. The final search was updated on May 24, 2026. Search terms included acupuncture, electroacupuncture, traumatic brain injury, brain injury, mitochondrial homeostasis, mitochondrial dynamics, mitophagy, mitochondrial biogenesis, oxidative stress, calcium signaling, and mitochondrial transfer. Studies were included when they reported acupuncture-related interventions and mitochondrial or mitochondria-associated outcomes in TBI or related brain injury models. Studies without mechanistic endpoints or relevance to mitochondrial regulation were excluded from the mechanistic synthesis. To avoid overgeneralization, the evidence was classified as direct TBI evidence, indirect evidence from related brain injury models, or hypothesis-generating evidence.

## Mitochondrial structural homeostasis

Mitochondrial dynamics serve as a core mechanism for maintaining mitochondrial structural homeostasis. Through the continuous processes of fission and fusion, mitochondria regulate mitochondrial morphology and functional stability. The balance between these processes directly determines the efficiency of mitochondrial energy metabolism and the capacity for damage repair. Under normal physiological conditions, mitochondrial fusion helps maintain membrane integrity and enables functional complementarity, while fission facilitates the clearance of damaged mitochondria and optimizes their intracellular distribution. The dynamic equilibrium between fission and fusion is essential for preserving mitochondrial homeostasis [12].

Mechanical injury and secondary ischemia-hypoxia following TBI can disrupt the aforementioned balance, often manifesting as excessive fission and reduced fusion, ultimately leading to mitochondrial dysfunction [13]. Dynamin-related protein 1 (Drp1), a key protein regulating mitochondrial fission, is frequently activated through post-translational modifications such as phosphorylation after TBI. This activation promotes its translocation to the mitochondrial outer membrane, inducing excessive mitochondrial fission and generating numerous dysfunctional mitochondrial fragments. This cascade results in reduced ATP synthesis, increased cytochrome C (Cyt-c) release, and eventual activation of apoptotic pathways [14, 15]. Concurrently, TBI can downregulate the expression of mitochondrial fusion-related proteins, such as OPA1, thereby inhibiting mitochondrial fusion and further exacerbating structural damage to mitochondria [16].

Evidence linking acupuncture to mitochondrial fission–fusion regulation after TBI should be interpreted in a graded manner. Direct TBI studies mainly show that acupuncture can reduce histopathological injury and modulate mitochondrial- or oxidative stress-related injury markers [9, 10, 17]. Related hemorrhagic brain injury evidence further suggests potential mitochondrial structural protection [18]. These findings support a structural protective effect but do not, by themselves, identify the full molecular pathway. More detailed evidence for Drp1, Fis1, Mfn2, and OPA1 regulation comes largely from cerebral ischemia–reperfusion or MCAO models, in which electroacupuncture reduced Drp1/Fis1 expression and mitochondrial translocation while increasing Mfn2/OPA1 expression, thereby improving mitochondrial morphology and neuronal survival [19, 20]. Because ischemic and traumatic injuries share secondary mitochondrial stress but differ in primary mechanical injury, these findings should be regarded as indirect support for a plausible mechanism rather than direct proof in TBI. Future TBI studies should combine ultrastructural analysis with Drp1 phosphorylation, mitochondrial translocation assays, fusion-protein quantification, and pathway-specific inhibition to determine whether acupuncture directly restores fission–fusion balance after mechanical brain injury.

## Mitochondrial quantity homeostasis

The maintenance of mitochondrial quantity homeostasis depends on a dynamic balance between mitochondrial biogenesis and the clearance of damaged mitochondria through mitophagy. After TBI, this balance may be disrupted by impaired mitochondrial biogenesis, defective autophagic flux, accumulation of dysfunctional mitochondria, and excessive or insufficient mitophagy depending on injury stage and cellular context. Current studies suggest that acupuncture may influence both sides of this generation-clearance balance [18, 21–23]. However, the strength of evidence differs substantially across mechanisms: some findings are directly derived from TBI or mTBI models, whereas others are inferred from ischemic or hemorrhagic brain injury. This section therefore distinguishes direct evidence from supportive indirect evidence and highlights where mechanistic validation in TBI is still required.

### Mitochondrial biogenesis

Mitochondrial biogenesis is a complex process that involves high-level coordination between the nuclear genome and the mitochondrial genome. Its core regulatory pathway is the PGC-1α/NRF1/TFAM signaling axis. Peroxisome proliferator-activated receptor gamma coactivator-1α (PGC-1α), as the central regulator of this pathway, first forms a complex by binding with nuclear receptors such as PPARγ and ERRα. It then synergistically enhances the transcriptional activity of nuclear respiratory factor-1 (NRF1), which subsequently upregulates the expression of mitochondrial transcription factor A (TFAM) [24, 25]. TFAM subsequently translocates into the mitochondria, where it directly governs the replication, maintenance, and transcription of mitochondrial DNA (mtDNA), serving as the key executor in initiating and completing mitochondrial biogenesis [26]. Ischemia-hypoxia in brain tissue following TBI significantly suppresses the activity of the PGC-1α/NRF1/TFAM pathway. This leads to a reduction in mitochondrial biogenesis, decreased mtDNA copy number, and impaired generation of new mitochondria, ultimately failing to compensate for the loss of mitochondrial quantity caused by the injury [27, 28].

Evidence for acupuncture-related promotion of mitochondrial biogenesis after TBI is suggestive but not yet comprehensive. A study of bloodletting at the twelve Jing-well points in TBI rats reported increased PGC-1α and TFAM expression and elevated mtDNA copy number 72 h after intervention, supporting a direct link between an acupuncture-related intervention and mitochondrial biogenesis in TBI [21]. In a mild TBI rat model, electroacupuncture improved Morris water maze performance and was associated with activation of the SIRT1/PGC-1α pathway, increased mitochondrial respiratory chain complex I activity, and higher ATP levels [29]. These studies support a potential role for acupuncture in restoring the mitochondrial generation arm after TBI. By contrast, evidence involving CB1R-dependent PGC-1α activation or synchronized upregulation of the PGC-1α/NRF1/TFAM axis is largely derived from non-TBI models and should be considered supportive only [22, 23]. Future studies should test whether pharmacological blockade or knockdown of PGC-1α, NRF-1, TFAM, SIRT1, or CB1R abolishes acupuncture-induced mitochondrial biogenesis and functional recovery in standardized TBI models.

### Mitophagy

Mitophagy is a quality control process that selectively clears damaged or redundant mitochondria, which is crucial for maintaining the integrity of the mitochondrial pool [30]. In the complex pathological environment of TBI, mitophagy plays a dual role: when moderately activated, it can promptly remove dysfunctional mitochondria, reduce ROS generation, inhibit the opening of the mitochondrial permeability transition pore (mPTP), prevent cytochrome c leakage, thereby exerting endogenous neuroprotective effects [31, 32]. On the other hand, excessive mitophagy or blocked autophagic flux can lead to massive accumulation of damaged mitochondria, exacerbating oxidative stress and energy metabolism disorders, and even interacting with other forms of cell death such as pyroptosis and ferroptosis, forming a complex injury network [33]. For example, in a TBI model, one study found that lactylation at the K286 site of the mitochondrial translation elongation factor Tufm post-TBI could inhibit Tufm-mediated mitophagy, making neurons more susceptible to apoptosis [34]. Another study suggested that copper ion accumulation may impair synaptic function by interfering with BNIP3-mediated mitophagy [35]. In summary, mitophagy possesses both protective and detrimental roles in TBI, and its function depends on "moderate activation" and "pathway integrity."

In mammalian cells, the PINK1/Parkin signaling pathway is the most extensively studied mechanism of mitophagy [36]. In TBI and related brain injury models, changes in PINK1/Parkin activity can influence mitochondrial quality control and neuronal fate [15, 37–39]. However, direct evidence that acupuncture regulates PINK1/Parkin-mediated mitophagy specifically after TBI remains insufficient. Most detailed evidence comes from cerebral ischemia–reperfusion models, in which electroacupuncture increased PINK1 and Parkin expression, promoted Parkin recruitment to damaged mitochondria, regulated p62 and LC3-II, reduced Rab7 accumulation, and partially restored LAMP-2-associated lysosomal function [18, 39]. These findings are valuable because TBI and ischemic injury share mitochondrial stress and autophagic flux impairment, but they should not be generalized as established TBI mechanisms. In TBI models, electroacupuncture has been reported to modulate IL-10 and AMPK/mTOR-related autophagy and energy markers [40], yet whether this reflects selective mitophagy rather than broader autophagy requires further validation using mitochondrial reporters, lysosomal flux assays, and PINK1/Parkin loss-of-function strategies.

In addition to the classical PINK1/Parkin pathway, receptor-mediated mitophagy involving FUNDC1 may also be relevant under hypoxic or mitochondrial stress conditions [41, 42]. At present, however, there is no direct evidence that acupuncture regulates FUNDC1-mediated mitophagy after TBI. Electroacupuncture has been shown to regulate the mTOR-ULK1/FUNDC1 axis in cerebral ischemia–reperfusion injury [41], but this should be treated as indirect evidence. Given that TBI includes both primary mechanical injury and secondary ischemia-hypoxia, FUNDC1 may represent a testable hypothesis rather than an established mechanism. Future studies should determine whether acupuncture alters FUNDC1 phosphorylation status, LC3 binding, mitochondrial recruitment, and mitophagic flux in TBI models at defined post-injury stages.

It is also important to emphasize that mitophagy regulation is not limited to protein signaling. Lipid metabolite-mediated mitochondrial quality control provides a novel conceptual framework for understanding long-term mitochondrial homeostasis after brain injury. For example, reduced p17/C18-ceramide transporter function and CerS1 expression have been linked to impaired ceramide-mediated autophagic defense mechanisms in chronic traumatic encephalopathy [43]. However, whether acupuncture regulates ceramide-dependent mitochondrial quality control after TBI has not been directly examined. Therefore, lipid-mediated mitophagy should be presented as an emerging hypothesis rather than an established acupuncture mechanism. Future work should combine lipidomics, mitochondrial subfraction analysis, and pathway-specific manipulation to test whether acupuncture affects ceramide distribution, mitochondrial lipid microdomains, and mitophagy selectivity after TBI.

## Functional and metabolic homeostasis

Mitochondrial function and metabolic homeostasis form the core foundation for maintaining cellular life activities, encompassing energy metabolism, redox balance, calcium homeostasis, and mitochondrial membrane potential stability, among other aspects. Following TBI, this precisely regulated homeostatic system is severely disrupted, primarily manifested as impaired energy metabolism, excessive ROS generation, Ca^2^⁺ homeostasis imbalance, and collapse of the mitochondrial membrane potential, ultimately damaging or even leading to neuronal death [4, 44]. Available preclinical evidence suggests that acupuncture may improve mitochondrial function and help re-establish metabolic homeostasis through multiple pathways, thereby potentially supporting neural repair after TBI.

### Energy metabolism

TBI can induce comprehensive impairment of mitochondrial energy metabolism. Specifically, this is reflected in decreased activity of mitochondrial respiratory chain complexes (such as succinate dehydrogenase SDH, NADH dehydrogenase, cytochrome c oxidase COX, etc.) alongside weakened Na⁺/K⁺-ATPase function, leading to reduced ATP production, imbalanced energy metabolism, and ultimately severe impacts on neuronal function and survival [45, 46]. Available preclinical studies suggest that acupuncture may ameliorate post-TBI energy metabolic disturbance. Several early classic acupuncture studies reported that in cerebral hemorrhage or injury models, electroacupuncture could enhance mitochondrial SDH and Na⁺/K⁺-ATPase activity while reducing lactic acid accumulation, thereby improving energy metabolism [47]. For example, in a mild TBI rat model, electroacupuncture increased ATP content and mitochondrial complex I activity, while upregulating the expression of the SIRT1/PGC-1α pathway [29]—both key molecules regulating mitochondrial biogenesis and energy metabolism. A study by Mu et al., using a rat model of cerebral ischemia–reperfusion injury, found that acupuncture at the "Baihui" (GV20) and "Dazhui" (GV14) acupoints upregulated the expression and activity of Na⁺/K⁺-ATPase, maintaining the intracellular/extracellular ion concentration gradient and membrane potential stability, alleviating cellular edema, and creating a stable environment for mitochondrial energy metabolism [48]. Taken together, these studies suggest, from different perspectives, that acupuncture modulate mitochondrial energy metabolism through multiple targets, potentially providing energetic support for post-TBI neuronal repair and functional recovery.

### Oxidative stress

Following TBI, impaired mitochondrial energy metabolism leads to excessive ROS production, coupled with a compromised endogenous antioxidant defense system, collectively triggering a severe oxidative stress response. As the core mediator of oxidative stress, ROS can attack mitochondrial membrane lipids, proteins, and mtDNA, exacerbating mitochondrial damage and creating a self-perpetuating vicious cycle of "oxidative stress-mitochondrial injury" [49, 50]. Under physiological conditions, antioxidant enzymes such as superoxide dismutase (SOD), glutathione peroxidase (GSH-Px), and catalase (CAT) within the body scavenge excess ROS to maintain redox balance. However, TBI leads to a decrease in antioxidant enzyme activity and a massive accumulation of ROS, resulting in elevated levels of the lipid peroxidation product malondialdehyde (MDA), which further disrupts mitochondrial function [50, 51].

Direct TBI evidence indicates that electroacupuncture can reduce oxidative stress-related markers and improve neurological outcomes. Liu et al. [44] reported that electroacupuncture decreased ROS, lipid peroxidation, MDA, Cyt-C, caspase-3, and PANX1-related signaling in TBI rats, suggesting regulation of a PANX1/ATP/Ca^2^⁺ pathway. Other acupuncture-related studies in TBI or related neurological conditions also suggest anti-inflammatory or antioxidant effects, although mitochondrial specificity remains insufficiently established [8, 52, 53]. Nevertheless, oxidative stress is a broad secondary injury process, and not all studies directly distinguish mitochondrial ROS from total cellular ROS. Therefore, future TBI studies should incorporate mitochondria-specific ROS indicators, respiratory chain assays, mitochondrial membrane potential measurements, and antioxidant pathway inhibition to clarify the extent to which acupuncture directly targets mitochondrial redox homeostasis.

### Calcium homeostasis, mPTP, and apoptosis

The disruption of calcium homeostasis constitutes a core event in secondary mitochondrial injury following TBI, and its pathological progression involves a network of interacting pathways. Among these pathways, ischemia-hypoxia-induced impairment of energy metabolism can lead to decreased Na⁺/K⁺-ATPase activity → accumulation of cytosolic Na⁺ → reverse-mode operation of the Na⁺/Ca^2^⁺ exchanger → massive Ca^2^⁺ influx → mitochondrial calcium overload, which forms a classic cascade reaction [54–56]. Mitochondrial Ca^2^⁺ overload further inhibits ATP synthesis, thereby establishing a vicious cycle of "energy failure-calcium overload" [57]. Moreover, the primary mechanical shear force of TBI can directly compromise the integrity of the cell membrane, resulting in energy-independent pathological Ca^2^⁺ influx. Meanwhile, glutamate excitotoxicity activates NMDA receptors, mediating excessive opening of Ca^2^⁺ channels, which acts synergistically with the aforementioned pathways to exacerbate mitochondrial calcium overload [56]. Notably, mPTP opening acts as a critical “molecular switch” linking calcium overload to mitochondrial membrane potential collapse, while its regulatory protein Cyp-D contributes to neuronal fate determination under mitochondrial stress.

**Fig. 1:**
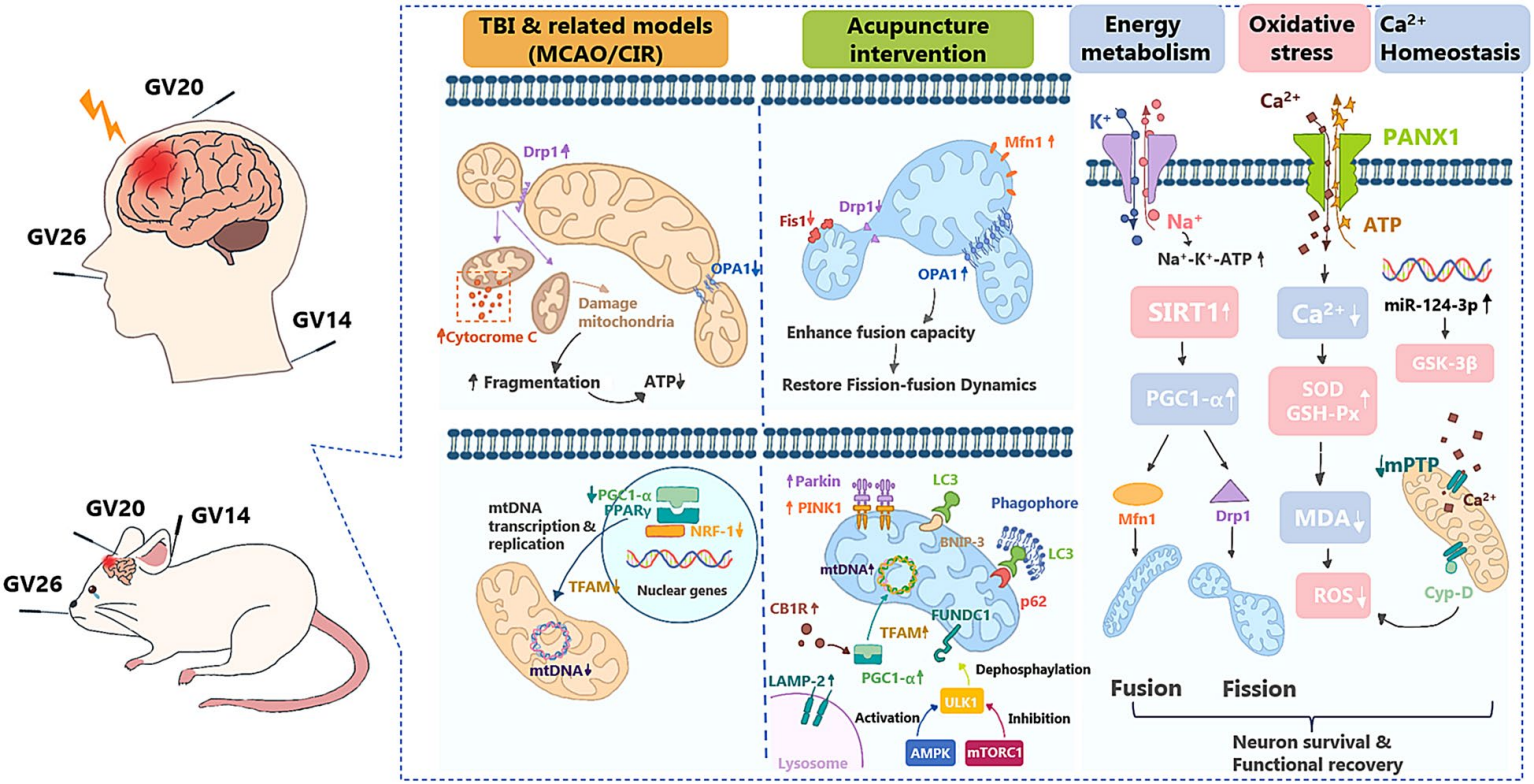
Integrated mechanistic overview of acupuncture-mediated regulation of mitochondrial homeostasis after TBI and related brain injury models. This figure was created by the authors. Because not all pathways shown have been directly validated in TBI models, this figure should be interpreted as an integrated mechanistic overview rather than a definitive TBI-specific pathway map.

Acupuncture-related regulation of the calcium homeostasis-membrane potential-apoptosis axis should also be interpreted cautiously. In TBI models, electroacupuncture has been associated with downregulation of Cyt-C, caspase-3, and caspase-9, as well as reductions in PANX1/ATP/Ca^2^⁺ signaling [44, 58]. These findings support a relationship between acupuncture, calcium-related signaling, mitochondrial apoptotic execution, and neuronal protection. However, several mechanistic links, including miR-124-3p/GSK-3β/Cyp-D-mediated mPTP regulation, are primarily supported by cerebral ischemia studies [59]. Likewise, preliminary evidence regarding astrocytic Connexin43 phosphorylation after mild TBI suggests a possible membrane-channel mechanism [60]. Broader acupuncture-related Cx43 literature remains indirect and should not be interpreted as TBI-specific mitochondrial evidence [61]. Accordingly, the calcium-mPTP-apoptosis axis is presented here as a plausible and partially supported mechanism that requires pathway-specific validation in TBI models (Fig. 1).

**Fig. 2:**
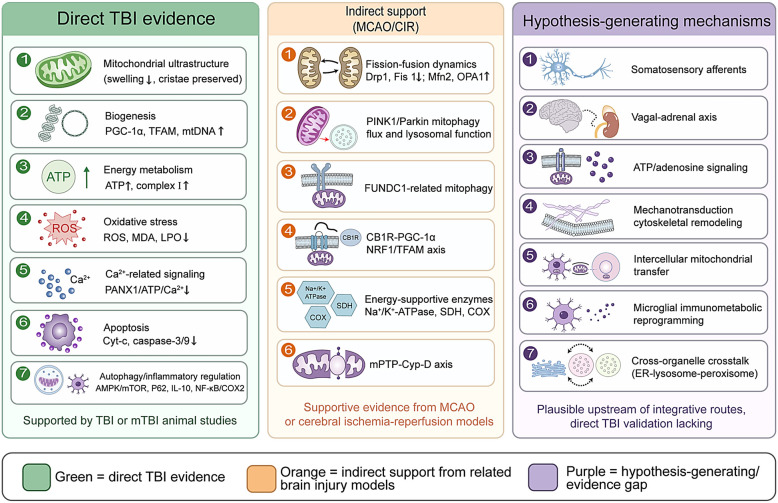
Evidence map of acupuncture-mediated mitochondrial regulation after TBI. This figure was created by the authors. This figure summarizes the evidence into three levels: direct TBI evidence, indirect support from related brain injury models, and hypothesis-generating mechanisms. It highlights established observations, supportive extrapolations from MCAO and CIR models, and unresolved mechanistic gaps

## Intercellular and microenvironmental regulation

The pathological alterations following TBI are not confined to mitochondrial damage within individual neurons. They also involve interactions among neurons, astrocytes, microglia, endothelial cells, and extracellular matrix components, together with mechanical disruption of axons, membranes, and cytoskeletal structures. Therefore, mitochondrial homeostasis after TBI should be viewed not only as a cell-autonomous process but also as part of a multicellular injury microenvironment. The following sections discuss intercellular mitochondrial transfer, immune-metabolic regulation, and cytoskeletal repair as emerging conceptual extensions. Because direct evidence for several of these mechanisms in acupuncture-treated TBI remains limited, they are presented as hypothesis-generating frameworks rather than established therapeutic pathways.

### Intercellular mitochondrial transfer

The traditional view holds that mitochondrial homeostasis primarily relies on cell-autonomous mechanisms such as autophagy, dynamics regulation, and biogenesis. However, recent research has unveiled "intercellular mitochondrial transfer" as a significant mechanism for maintaining central nervous system homeostasis. Particularly under the pathological condition post-TBI, where neuronal energy demands surge dramatically while endogenous mitochondria are severely impaired, astrocytes can transfer functional mitochondria or mitochondrial components to damaged neurons through tunneling nanotubes (TNTs), Cx43-associated gap junction structures, or extracellular vesicles. This process rapidly restores neuronal energy metabolism and inhibits apoptosis [62, 63]. In vitro models simulating TBI injury, Ren et al. [64] found that the GJA1-20K subtype, specifically expressed in astrocytes, enhances the efficiency of Cx43-dependent mitochondrial transfer, thereby promoting the recovery of energy metabolism and neurite regeneration in injured neurons. Furthermore, multiple studies and reviews have highlighted that astrocyte-to-neuron mitochondrial transfer under stress conditions such as brain injury represents a rapid, non-canonical, yet highly adaptive neuroprotective mechanism. This provides a novel explanatory framework for understanding how neural network homeostasis and cell survival are maintained after TBI [65–67].

Intercellular mitochondrial transfer represents an emerging and highly exploratory research direction in neurotrauma. Current evidence suggesting that acupuncture may influence astrocyte-neuron mitochondrial transfer is preliminary and mainly derived from ischemic stroke models. Therefore, whether this mechanism substantially contributes to acupuncture-mediated neuroprotection after TBI remains to be validated. In models of acute cerebral ischemia, Guo et al. [68] reported that electroacupuncture promoted mitochondrial transfer from astrocytes to neurons, potentially through the CD38-cADPR-Ca^2^⁺ axis, tunneling nanotube formation, and enhanced mitochondrial transport capacity. These findings provide a biologically plausible hypothesis for acupuncture-mediated intercellular support after brain injury, but they do not yet establish a TBI-specific mechanism. Future studies should employ mitochondrial lineage tracing, astrocyte-specific CD38 or Cx43 manipulation, and TBI models with defined injury severity to test whether acupuncture truly mobilizes astrocyte-to-neuron mitochondrial transfer after mechanical brain injury.

### Immune microenvironment

A close bidirectional regulatory relationship exists between mitochondrial function and neuroinflammation. Following TBI, the mitochondrial metabolic pattern of microglia undergoes reprogramming, shifting from oxidative phosphorylation to glycolysis, which drives their polarization towards the pro-inflammatory M1 phenotype [69]. This leads to the release of large amounts of inflammatory factors, further exacerbating neuronal mitochondrial damage [70, 71]. By modulating microglial activation and inflammatory signaling, acupuncture may indirectly reshape the immune microenvironment [72, 73].

Several studies indicate that acupuncture can modulate microglial activation and inhibit inflammatory signaling, including NF-κB/COX2-related pathways [53, 72, 73], while immunometabolic evidence from CNS injury research provides broader conceptual support for microglia-mitochondria crosstalk [74]. These effects may indirectly protect neuronal mitochondria by reducing exposure to IL-1β, TNF-α, ROS, and other inflammatory mediators. However, evidence that acupuncture directly reprograms microglial mitochondrial metabolism after TBI remains limited. Future experiments should measure microglial oxidative phosphorylation, glycolysis, mitochondrial membrane potential, and cell-specific mitochondrial ROS, ideally using single-cell or cell-sorted approaches. Such studies would clarify whether acupuncture regulates an immune-mitochondrial crosstalk pathway or primarily exerts broader anti-inflammatory effects.

### Cytoskeletal remodeling

When elucidating the mechanisms by which acupuncture modulates mitochondrial function, it is essential to fully recognize the pathological differences between TBI and other central nervous system injuries such as ischemic stroke. Although both conditions involve secondary mitochondrial damage mediated by ischemia and hypoxia, TBI is characterized by unique primary mechanical injury features. The shear forces generated by external impact can directly induce changes in mitochondrial membrane permeability, disruption of cristae structure, and interruption of axonal transport. This physical damage typically precedes metabolic dysfunction and inflammatory responses, making structural repair of mitochondria particularly critical [75, 76].

Mechanical repair and cytoskeletal remodeling represent a plausible but still insufficiently validated framework for acupuncture-mediated neuroprotection after TBI. Studies outside or adjacent to TBI contexts suggest that acupuncture-related mechanical stimulation can influence cytoskeletal rearrangement, membrane repair, mechanosensitive channels such as Piezo1 and TRP channels, and integrin-related signaling pathways [77–80]. Because TBI is characterized by primary shear stress, axonal transport interruption, and membrane disruption, these pathways may be relevant to mitochondrial positioning and axonal mitochondrial trafficking. Nevertheless, a direct causal chain from acupuncture stimulation to cytoskeletal repair and mitochondrial redistribution in TBI has not been demonstrated. Future studies should assess axonal microtubule integrity, mitochondrial transport directionality, membrane resealing, and mechanotransduction blockade to determine whether this mechanism contributes to functional recovery.

To systematically present the evidence while avoiding overgeneralization, Table 1 classifies studies according to whether they provide direct TBI evidence, indirect supportive evidence from related brain injury models, or hypothesis-generating evidence. This structure is intended to clarify the evidentiary basis for each proposed mechanism and to highlight the translational gaps that remain.

**Table 1:** Evidence grading of acupuncture-mediated mitochondrial regulation in TBI and related brain injury models

Refs	Evidence category/model	Intervention/parameters	Timing/outcome point	Mitochondrial endpoints	Interpretation/limitation
Li et al. (2024) [17]	Direct evidence: SD rat TBI	EA: GV20, GV26, ST36, PC6; 1–2 mA; 1/3 Hz; 15 min	Post-injury treatment for 7 days; assessment after treatment	Mitochondrial swelling and outer membrane rupture decreased; cristae visibility improved	Supports structural protection in TBI, but molecular fission–fusion pathways were not fully dissected
Shen et al. (2018) [21]	Direct evidence: SD rat TBI	Pricking blood at twelve Jing-well points; 10 μL per acupoint	Once every 12 h for 3 days; reported at 72 h	PGC-1α↑, TFAM↑, mtDNA copy number↑	Supports mitochondrial biogenesis in TBI; modality differs from EA and requires replication
Jin et al. (2024) [29]	Direct evidence: SD rat mTBI	EA: GV20, EX-HN3; 2 mA; 4/20 Hz; 15 min	Post-injury treatment for 7 days; assessment after treatment	SIRT1/PGC-1α↑, mitochondrial respiratory chain complex I↑, ATP↑	Supports a biogenesis/energy-metabolism mechanism in mTBI
Wu et al. (2023) [40]	Direct evidence: SD rat TBI	EA: LI11, LI4, GV20, CV4, ST36; 1 mA; 1 Hz; 15 min	Post-injury treatment for 14 days; assessment after treatment	IL-10↑, AMPK/mTOR signaling regulated, ATP↑, ADP/AMP↓, p62↑	Supports autophagy/energy regulation in TBI; selective mitophagy requires further validation
Liu et al. (2025) [44]	Direct evidence: SD rat TBI	EA: LI11, LI4, GV20, CV4, ST36, KI1; 1 mA; 1 Hz; 15 min	Post-injury treatment for 14 days; assessment after treatment	PANX1/ATP/Ca^2^⁺↓, Cyt-C↓, caspase-3↓, ROS↓, LPO↓, MDA↓	Supports regulation of oxidative stress, calcium-related signaling, and apoptosis in TBI
Gu et al. (2020) [58]	Direct evidence: SD rat TBI	EA/MA: GV20, GV26, PC6, ST36; 2 Hz; current intensity not reported; 10 min plus 30 s MA at GV26	Post-injury treatment for 14 days; assessment after treatment	Cyt-C↓, caspase-9↓	Supports anti-apoptotic effects; incomplete reporting of EA intensity limits reproducibility
Zhang et al. (2023) [53]	Direct evidence: SD rat TBI	EA or MA: GV26, GB20, PC6; EA 2 mA; 2/100 Hz; 15 min	Post-injury treatment for 7 days; assessment after treatment	ROS↓, NF-κB↓, COX2↓, IL-6↓, IL-1β↓, TNF-α↓, Arg-1↑, IL-10↑	Supports anti-inflammatory/oxidative regulation; mitochondrial specificity is indirect
Jia et al. (2023) [11]	Clinical evidence: human mTBI	EA: PC6, GV26; 0.5–5 mA; 2/100 Hz; 30 min	Clinical treatment for 14 days; assessment after treatment	Cognitive score↑; NSE↓, GFAP↓, HIF-1α↓, MDA↓	Suggests clinical benefit in mTBI, but mitochondrial biomarkers and large multicenter trials are lacking
Li et al. (2024) [20]	Indirect evidence: SD rat MCAO	EA: GV20, GV14; 1 mA; 2/5 Hz; 20 min	Treatment for 7 days	Drp1↓, Fis1↓, Mfn2↑, OPA1↑, mitochondrial fragmentation alleviated	Supports a plausible fission–fusion mechanism; not TBI-specific
Li et al. (2025) [19]	Indirect evidence: SD rat MCAO	EA: LI11, ST36; 1 mA; 4/20 Hz; 30 min	Treatment for 7 days	Drp1↓, OPA1↑, mitochondrial distribution improved	Supports mitochondrial dynamics regulation in ischemic injury only
Sun et al. (2021) [23]	Indirect evidence: C57BL/6J mouse MCAO	EA pretreatment: GV20; 1 mA; 2/15 Hz; 30 min	Pretreatment before ischemic modeling	CB1R/PGC-1α/NRF1/TFAM axis↑, Cyt-C↓, ROS↓, mPTP↓	Suggests an upstream CB1R-PGC-1α pathway; needs TBI validation
Wang et al. (2019) [39]	Indirect evidence: SD rat MCAO	EA: GV20, ST36; 1 mA; 20 Hz; 30 min	Post-ischemia treatment; outcome timing as reported in source	PINK1/Parkin↑, LC3↑, MMP↑, ATP↑, mPTP↓, ROS/MDA/iNOS/3-NT↓, Rab7↓, LAMP-2↑	Supports mitophagy and autophagic flux regulation in ischemia; not direct TBI evidence
Tian et al. (2022) [41]	Indirect evidence: SD rat MCAO	EA pretreatment: GV20, GV26; 1 mA; 2/50 Hz; 30 min	Pretreatment for 5 days	mTOR-ULK1/FUNDC1 pathway regulated; MMP↑	Supports FUNDC1-related hypothesis; TBI-specific evidence lacking
Bao et al. (2008) [47]	Indirect evidence: Wistar rat ICH	EA/scalp acupuncture: GV20, EX-HN5; 1 mA; 2 Hz; 30 min	Treatment for 7 days	SDH↑, COX↑, ATP↑, lactic acid↓	Supports energy metabolism regulation in hemorrhagic injury; indirect for TBI
Mu et al. (2009) [48]	Indirect evidence: SD rat MCAO	Manual plus EA: GV20, GV14; 1–2 mA; 80–100 Hz; twirling 0.5 min plus 60-min EA	Post-ischemia treatment; outcome timing as reported in source	Na⁺/K⁺-ATPase↑, SOD↑, LPO↓, MDA↓	Supports ion pump/oxidative stress regulation; indirect for TBI
Guo et al. (2025) [68]	Hypothesis-generating evidence: SD rat tMCAO	EA: GV20, PC6, SP6; 2 mA; 2/10 Hz	Pretreatment or 12 h post-modeling intervention	CD38-cADPR-Ca^2^⁺ pathway regulated; TNT-related proteins↑; mitochondrial transfer↑, MMP↑, ATP↑	Supports mitochondrial-transfer hypothesis in ischemia; TBI validation required
Zhang et al. (2023) [59]	Indirect evidence: SD rat MCAO	EA pretreatment: GV20, GV16, GV14; 1–2 mA; 2/15 Hz; 20 min	Pretreatment for 7 days	mPTP↓, GSK-3β↓, Cyp-D↓, Cyt-C↓, caspase-3↓	Supports mPTP-apoptosis axis in ischemia; indirect for TBI

## Evidence integration and future directions

### Acupoint and parameter specificity

Acupoint specificity and stimulation parameters are important but remain insufficiently resolved in the current literature. Across the studies summarized in Table 1, commonly used acupoints include GV20, GV26, GV14, GV16, ST36, PC6, LI4, and LI11. Among them, GV20, GV26, GV14, and GV16 appear repeatedly in acupuncture prescriptions for TBI or related brain injury models, indicating a recurring empirical preference for Governor Vessel acupoints in brain-related conditions. This preference is partly supported by general evidence on acupoint specificity [81], studies showing that Governor Vessel acupoints may influence cerebral hemodynamic regulation [82], and neurophysiological discussions linking scalp acupuncture to cerebral blood flow regulation [83]. In TBI models, acupuncture prescriptions including Governor Vessel acupoints have also been associated with reduced microglial activation or neural repair-related outcomes [73, 84]. However, these studies do not establish Governor Vessel-specific mitochondrial regulation after TBI. Direct comparisons between Governor Vessel acupoints, non-Governor Vessel acupoints, non-acupoints, and sham stimulation remain insufficient, especially when mitochondrial endpoints are used as primary outcomes. Therefore, the frequent use of Governor Vessel acupoints should be interpreted as a recurring empirical and neurophysiologically plausible pattern rather than proof that Governor Vessel stimulation is superior for mitochondrial regulation after TBI. Many studies vary simultaneously in model type, injury severity, acupoint selection, frequency, intensity, duration, treatment window, and outcome time point, making it difficult to isolate the effect of any single parameter.

A similar caution applies to frequency dependency. Based on the studies summarized in Table 1, several tentative parameter–effect associations can be observed, but none should be regarded as established rules. For example, a 20 Hz EA protocol in an ischemia–reperfusion model was associated with PINK1/Parkin-related mitophagy and autophagic flux regulation [39], whereas 1 Hz EA protocols in TBI models were associated with autophagy/energy regulation and PANX1/ATP/Ca^2^⁺ signaling [40, 44]. Mixed-frequency protocols, such as 2/5 Hz or 4/20 Hz, have also been reported in studies involving mitochondrial dynamics, mitochondrial biogenesis, or energy metabolism [20, 29]. However, these associations are confounded by differences in disease model, acupoint combination, stimulation intensity, treatment duration, and outcome timing. They should therefore be considered hypothesis-generating parameter–effect patterns rather than frequency-specific conclusions. Future studies should adopt factorial experimental designs that independently vary frequency, intensity, acupoint selection, and treatment timing within the same TBI model.

Temporal dynamics should also be considered. Pretreatment studies in MCAO or cerebral ischemia–reperfusion models mainly provide indirect support for mechanisms related to CB1R–PGC-1α signaling, FUNDC1-related mitophagy, mPTP regulation, or mitochondrial transfer [23, 41, 59, 68]. In contrast, post-injury TBI or mTBI studies provide more direct evidence for effects on mitochondrial ultrastructure, mitochondrial biogenesis, energy metabolism, oxidative stress, calcium-related signaling, apoptosis, and selected autophagy-related pathways [17, 21, 29, 40, 44, 58]. A cautious working hypothesis is that acute post-TBI interventions may prioritize limiting oxidative stress, calcium overload, excessive mitochondrial fission, and apoptosis, whereas subacute interventions may focus on restoring mitochondrial biogenesis, balanced mitophagy, and energy metabolism. This stage-dependent strategy remains speculative and should be tested in standardized TBI models using factorial designs that compare acupoint selection, sham/non-acupoint controls, stimulation frequency, intensity, duration, and treatment window.

Despite encouraging findings, several limitations should be explicitly acknowledged. First, a considerable proportion of mechanistic studies cited in this review are derived from ischemic stroke, cerebral hemorrhage, or other non-TBI models. Although these disorders share secondary injury mechanisms with TBI, including oxidative stress, mitochondrial dysfunction, calcium overload, and neuroinflammation, they differ substantially in primary pathological features such as mechanical shear injury, diffuse axonal damage, membrane disruption, and cytoskeletal damage. Therefore, extrapolation of non-TBI findings to TBI should be conservative.

Second, most available studies are small-scale animal experiments with heterogeneous modeling methods, acupuncture protocols, and outcome measures. Reporting is sometimes incomplete, particularly with respect to stimulation intensity, sham controls, allocation concealment, blinding, and precise outcome time points. These limitations reduce reproducibility and cross-study comparability. Several mechanisms discussed in this review, including intercellular mitochondrial transfer, lipid-mediated mitophagy, and cytoskeletal repair, remain largely hypothetical in acupuncture-treated TBI and require direct validation.

Third, high-quality clinical evidence remains insufficient. Current clinical studies are limited by small sample sizes, heterogeneous acupuncture prescriptions, lack of standardized stimulation parameters, and limited use of mechanistic biomarkers. Therefore, the clinical translational value of acupuncture-mediated mitochondrial regulation in TBI requires validation through large-scale, multicenter, randomized controlled trials with clearly defined injury severity, treatment windows, sham controls, functional endpoints, and mitochondrial or neuroinjury biomarkers.

### Upstream neural and humoral pathways

Most current studies focus on downstream mitochondrial molecules such as Drp1, PINK1/Parkin, PGC-1α, TFAM, ROS, and mPTP, but the upstream pathways connecting peripheral acupuncture stimulation to intracranial mitochondrial responses remain poorly defined. Current neuroanatomical and physiological evidence suggests that electroacupuncture can activate somatosensory afferents and body-region-specific autonomic reflexes. For example, low-intensity electroacupuncture at hindlimb acupoints has been shown to recruit PROKR2-lineage sensory neurons and drive a vagal-adrenal anti-inflammatory axis [86]. Such peripheral-to-central neural transmission may engage spinal, brainstem, hypothalamic, and autonomic circuits that influence cerebral blood flow, neuroinflammation, and metabolic stress [83, 85, 86]. In parallel, autonomic and vagal anti-inflammatory pathways may regulate systemic inflammatory tone and thereby indirectly reduce inflammatory pressure on neuronal mitochondria [86]. At the local acupoint level, acupuncture has been shown to increase extracellular adenosine, and adenosine A1 receptor activation is required for part of its local biological effect in experimental models [87]. Therefore, ATP/adenosine, neuropeptides, and other locally released mediators may serve as humoral triggers that initiate downstream central immune-metabolic responses. Mechanical stimulation may also act through connective-tissue and cellular mechanotransduction: needle manipulation induces fibroblast cytoskeletal remodeling, supporting a mechanotransduction-based mechanism that may involve actin remodeling and calcium-dependent signaling [88].

However, direct evidence that these upstream neural, humoral, or mechanotransductive pathways are necessary for acupuncture-mediated mitochondrial regulation after TBI remains limited. Future studies should combine sensory nerve blockade, vagotomy, chemogenetic or optogenetic manipulation, receptor antagonists, and cell-specific mitochondrial readouts to identify which upstream circuits are required for mitochondrial regulation after TBI.

### Cross-organelle regulation

Although this review focuses on mitochondria, mitochondrial homeostasis after TBI is tightly coupled to other organelles. Endoplasmic reticulum (ER)-mitochondria contact sites, including mitochondria-associated membranes (MAMs), regulate Ca^2^⁺ transfer, lipid and metabolite exchange, mitochondrial dynamics, mPTP opening, apoptosis, inflammation, and autophagy-related signaling [89]. Experimental TBI evidence further supports this concept: severe TBI induces rapid reorganization of ER-mitochondrion contacts in the perilesional cortex, and modulation of ER-mitochondrion tethering can attenuate mitochondrial oxidative stress, ER stress-mediated apoptosis, neuroinflammation, and neurological deficits [90].

Lysosomes determine whether mitophagy proceeds to complete degradation or becomes stalled autophagic flux; dysregulated autophagy-lysosome function is therefore an important component of neurotrauma pathology and a potential therapeutic target [40, 91]. Peroxisomes participate in redox balance and lipid metabolism and interact functionally with mitochondria through shared control of fatty-acid metabolism, ROS handling, and organelle dynamics [92]. Lipid droplets may also participate in brain-injury responses: traumatic injury has been associated with lipid-droplet/TDP-43-positive microglial states and altered inflammatory resolution, suggesting that lipid storage organelles may influence neuroinflammation and repair [93].

Acupuncture may therefore regulate mitochondrial homeostasis indirectly through broader organelle networks rather than acting on mitochondria alone. This possibility remains hypothetical in acupuncture-treated TBI models and should be tested using live-cell imaging, spatial transcriptomics, proteomics, lipidomics, and organelle-specific reporters to clarify whether acupuncture coordinates mitochondria-ER-lysosome-peroxisome-lipid droplet interactions during different stages of TBI repair.

### Summary and outlook

This review summarizes the current evidence and mechanistic hypotheses regarding acupuncture-mediated regulation of mitochondrial homeostasis after TBI. Direct TBI studies support the possibility that acupuncture or electroacupuncture can improve mitochondrial ultrastructure, energy metabolism, oxidative stress balance, calcium-related signaling, apoptosis, and selected autophagy-related pathways. However, many detailed molecular mechanisms, including Drp1/Mfn2-mediated fission–fusion regulation, PINK1/Parkin or FUNDC1-mediated mitophagy, CB1R-PGC-1α-mediated biogenesis, intercellular mitochondrial transfer, ceramide-dependent mitophagy, and cytoskeletal repair, are supported mainly by indirect evidence or remain hypothesis-generating in the TBI context( Fig. 2 ).

Based on the available evidence, we propose a convergent working model in which acupuncture does not act through a single mitochondrial target, but may modulate a secondary injury network centered on mitochondrial stress. In this model, TBI-induced oxidative stress, calcium overload, energy failure, excessive mitochondrial fission, impaired mitophagy, mPTP opening, and apoptotic signaling form a self-amplifying pathological loop. Acupuncture may influence this loop through upstream neural, humoral, immune, and mechanotransductive pathways, thereby indirectly stabilizing mitochondrial structure, mitochondrial quality control, and metabolic function. This working model should be regarded as hypothesis-generating rather than definitive, because many detailed molecular links remain supported mainly by indirect or preclinical evidence.

It is therefore important to emphasize that mitochondrial structural, quantitative, and functional/metabolic homeostasis do not exist in isolation. Excessive oxidative stress may promote mitochondrial fission, fragmented mitochondria may activate mitophagy, impaired mitophagy may aggravate ROS generation and energy failure, and calcium overload may trigger mPTP opening and apoptosis. These processes interact with each other and may collectively amplify secondary neuronal injury after TBI. Acupuncture may exert protective effects by modulating several nodes within this interconnected network rather than by targeting a single pathway. At present, however, the exact upstream signals, temporal windows, cell-type specificity, and organelle crosstalk mechanisms remain incompletely defined.

Future research should focus on several priorities. First, standardized TBI models and reporting criteria are needed to improve reproducibility across studies. Second, systematic parameter-response studies should independently evaluate acupoint selection, stimulation frequency, intensity, duration, and timing. Third, mechanistic experiments should use pathway inhibition, genetic manipulation, cell-type-specific mitochondrial reporters, and mitochondrial functional assays to distinguish direct mitochondrial effects from broader anti-inflammatory or neuroprotective effects. Fourth, intercellular mitochondrial transfer and cross-organelle regulation should be tested with appropriate tracing and organelle-specific methods before being presented as established mechanisms. Finally, large-scale multicenter randomized controlled trials with sham controls, standardized protocols, clinically meaningful endpoints, and mitochondrial or neuroinjury biomarkers are required to determine whether acupuncture-mediated mitochondrial regulation has genuine translational value for TBI patients. Current findings provide a conceptual and experimental basis for future studies, but they do not yet establish acupuncture as a clinically validated mitochondrial therapy for TBI.

## Data Availability

No datasets were generated or analysed during the current study.

## Abbreviations

AMPK: AMP-activated protein kinase
Arg-1: Isoform 1 of arginase
BNIP3: BCL2/adenovirus E1B 19 kDa protein-interacting protein 3
cADPR: Cyclic adenosine diphosphate ribose
CB1R: Cannabinoid receptor 1
CD38: Cluster of differentiation 38
CIR: Cerebral ischemia–reperfusion injury
COX: Cytochrome C oxidase
CV: Conception vessel
Cyt-c: Cytochrome C
Cyp-D: Cyclophilin D
Drp1: Dynamin-related protein 1
EX-HN: Extra Points of Head and Neck (EX-HN3: Yintang; EX-HN5: Taiyang)
Fis1: Fission 1
FUNDC1: FUN14 domain containing 1
GSK-3β: Glycogen synthase kinase 3 beta
GV: Governor Vessel
GSH-Px: Glutathione peroxidase
ICH: Intracerebral hemorrhage
IL: Interleukin
iNOS: Inducible nitric oxide synthase
KI: Kidney Meridian (KI1: Yongquan)
LAMP-2: Lysosome-Associated Membrane Protein 2
LC3: Microtubule-Associated Protein 1 Light Chain 3
LI: Large Intestine Meridian (LI11: Quchi; LI4: Hegu)
LPO: Lipid peroxidation
MA: Manual acupuncture
MCAO: Middle cerebral artery occlusion
MDA: Malondialdehyde
Mfn1: Mitofusin 1
Mfn2: Mitofusin 2
miR-124-3p: MicroRNA-124-3p
mPTP: Mitochondrial permeability transition pore
MMP: Mitochondrial membrane potential
mtDNA: Mitochondrial DNA
mTORC1: Mammalian target of rapamycin complex 1
Na⁺/K⁺-ATPase: Sodium–Potassium ATPase
NF-κB: Nuclear Factor-Kappa B
NRF1: Nuclear Respiratory Factor 1
OPA1: Optic atrophy 1
PANX1: Pannexin 1
Parkin: E3 Ubiquitin Ligase Parkin
PC: Pericardium Meridian (PC6: Neiguan)
PGC-1α: Peroxisome proliferator-activated receptor gamma coactivator 1-alpha
PPARγ: Peroxisome proliferator-activated receptor gamma
PINK1: PTEN-Induced Kinase 1
p62: Sequestosome 1
Rab7: Ras-related protein Rab-7A
ROS: Reactive oxygen species
SDH: Succinate dehydrogenase
SIRT1: Sirtuin 1
SOD: Superoxide dismutase
SP: Spleen Meridian (SP6: Sanyinjiao)
ST: Stomach Meridian (ST36: Zusanli)
TFAM: Mitochondrial transcription factor A
TNF-α: Tumor necrosis factor-alpha
TNT: Tunneling nanotube
tMCAO: Transient middle cerebral artery occlusion
ULK1: Unc-51 like autophagy activating kinase 1
3-NT: 3-Nitrotyrosine
